# The Effect of Blindness on Long-Term Episodic Memory for Odors and Sounds

**DOI:** 10.3389/fpsyg.2018.01003

**Published:** 2018-06-20

**Authors:** Stina Cornell Kärnekull, Artin Arshamian, Mats E. Nilsson, Maria Larsson

**Affiliations:** ^1^Gösta Ekman Laboratory, Department of Psychology, Stockholm University, Stockholm, Sweden; ^2^Division of Psychology, Department of Clinical Neuroscience, Karolinska Institutet, Stockholm, Sweden; ^3^Center for Language Studies, Donders Institute for Brain, Cognition and Behaviour, Radboud University, Nijmegen, Netherlands

**Keywords:** blindness, compensatory effect, environmental sounds, episodic recognition, identification, long-term odor memory

## Abstract

We recently showed that compared with sighted, early blind individuals have better episodic memory for environmental sounds, but not odors, after a short retention interval (∼ 8 – 9 min). Few studies have investigated potential effects of blindness on memory across long time frames, such as months or years. Consequently, it was unclear whether compensatory effects may vary as a function of retention interval. In this study, we followed-up participants (*N* = 57 out of 60) approximately 1 year after the initial testing and retested episodic recognition for environmental sounds and odors, and identification ability. In contrast to our previous findings, the early blind participants (*n* = 14) performed at a similar level as the late blind (*n* = 13) and sighted (*n* = 30) participants for sound recognition. Moreover, the groups had similar recognition performance of odors and identification ability of odors and sounds. These findings suggest that episodic odor memory is unaffected by blindness after both short and long retention intervals. However, the effect of blindness on episodic memory for sounds may vary as a function of retention interval, such that early blind individuals have an advantage over sighted across short but not long time frames. We speculate that the finding of a differential effect of blindness on auditory episodic memory across retention intervals may be related to different memory strategies at initial and follow-up assessments. In conclusion, this study suggests that blindness does not influence auditory or olfactory episodic memory as assessed after a long retention interval.

## Introduction

In everyday life, accurate episodic memory (i.e., memory of specific events in time and space) is crucial. Whereas sighted individuals to some extent rely on visual cues for remembering different types of sensory information, blind individuals clearly cannot. Research has shown that visual deprivation may result in a more developed ability to remember non-visual information. For example, compensatory effects of blindness have been observed for episodic and short-term memory for verbal material that was read aloud (Röder et al., 2001; Amedi et al., 2003; Hötting and Röder, 2009; Occelli et al., 2013, 2016; Pasqualotto et al., 2013). Moreover, Röder and Rösler (2003) showed better episodic recognition for environmental sounds in congenitally blind individuals than in sighted (cf. Cobb et al., 1979). We recently corroborated these findings by demonstrating that blind participants, especially those blind since birth or early childhood, had better episodic recognition performance for environmental sounds than sighted (Cornell Kärnekull et al., 2016). Previous studies on blind and sighted individuals indicate that compensatory effects may be more pronounced for congenital or early onset blindness than late onset blindness (e.g., Röder and Rösler, 2003; Gougoux et al., 2004; Wan et al., 2010). The effect of onset age of blindness has been suggested to be related to different levels of neuroplasticity of the brain (see Merabet and Pascual-Leone, 2010 for a review).

In comparison to the auditory sense, research is relatively sparse regarding effects of blindness on olfactory functions (see Kupers and Ptito, 2014; Araneda et al., 2016 for reviews). Previous research has primarily assessed olfactory sensitivity, quality discrimination, and identification ability (Kupers and Ptito, 2014). Our previous study (Cornell Kärnekull et al., 2016) was one of the first to address whether there are compensatory effects of blindness on episodic olfactory memory. In contrast to the observed superiority in episodic auditory memory of early blind individuals, we showed no compensatory effects of blindness for this modality. However, episodic recognition was assessed approximately 8 – 9 min after encoding, which limits the generalizability of our results. It is well known that the time length that follows learning affects memory consolidation processes in both human and non-human animals (e.g., Frankland and Bontempi, 2005; Kandel et al., 2014). More specifically, whereas some consolidation processes take place shortly after learning, other processes are more gradual across time and involves reorganization of connected brain regions (Frankland and Bontempi, 2005). This could potentially implicate that effects of blindness on episodic memory performance vary as a function of the length of the retention interval and modality.

Hence, it is still unknown whether the observed superiority in auditory memory among the early blind individuals also would be prevalent after a considerably longer retention interval, such as approximately 1 year. Furthermore, although blindness previously showed no influence on olfactory memory, there could still be an effect at significantly longer time frames for this modality. Thus, studying the interaction between retention interval and memory performance is important, especially as our knowledge of compensatory effects of blindness on memory is almost exclusively based on studies using relatively short retention intervals (seconds: e.g., Raz et al., 2007; Pasqualotto et al., 2013, minutes: e.g., Röder et al., 2001; Röder and Rösler, 2003; Cornell Kärnekull et al., 2016; Occelli et al., 2016; days: Occelli et al., 2016, week: Occelli et al., 2016).

Hence, our knowledge about odor and sound memory across longer time intervals is derived from studies on sighted individuals. Here, studies in episodic olfactory memory indicate that common environmental odors are forgotten relatively slowly, when assessed across months (e.g., Lawless and Cain, 1975; Lawless, 1978; Murphy et al., 1991). Also, whereas common and familiar odors are better remembered than unfamiliar ones, the forgetting functions seems to be similar (Cornell Kärnekull et al., 2015). In contrast to olfactory studies, research on memory of environmental sounds in sighted has primarily focused on relatively short retention intervals, such as seconds (Lawrence and Banks, 1973), minutes up to an hour (Bartlett, 1977), or days (Bower and Holyoak, 1973; Lawrence et al., 1979). Moreover, few studies in this field have applied several retention intervals (e.g., Bartlett, 1977; Bigelow and Poremba, 2014; Lawrence et al., 1979), although Lawrence et al. (1979) showed that the effect of retention interval (immediate - 2 days - 7 days) was relatively modest.

To the best of our knowledge, the only memory study with blind and sighted individuals that has used longer time intervals is the study by Amedi et al. (2003): Episodic recognition for words was assessed 6 months after encoding and was shown to be better in blind than sighted participants. In the same study, brain activation in the primary visual brain area (V1) was positively correlated with memory performance among the blind but not the sighted participants, suggesting a functional role of V1 for verbal memory (Amedi et al., 2003). This finding nicely exemplifies cross-modal plasticity, which has been observed during the processing of various sensory stimuli (e.g., auditory) in blind individuals (e.g., Cecchetti et al., 2016). Thus, reorganization and cross-modal plasticity of the brain may be important factors for explaining why blindness might be related to better episodic memory for verbal material even after longer time intervals. Moreover, a number of studies have shown that blind individuals have better selective attention toward one sensory modality while ignoring task-irrelevant sensory stimuli (Pasqualotto and Proulx, 2012; Occelli et al., 2013). Although this finding is suggested to be a consequence of a less developed multimodal sensory integration ability among blind individuals (Pasqualotto and Proulx, 2012), better selective attention may have a positive influence on memory ability. However, it is not known if better selective attention to one sense only benefits the tasks at hand, such as recognition for auditory events in the short-term, or if it also benefits memory over longer time. Taken together, more studies and knowledge about memory performance under different conditions are required to be able to understand the mechanisms that modulate the observed differences in episodic memory between blind and sighted individuals. Moreover, episodic memory assessed across longer time intervals is not least valuable from an ecological point of view considering that in everyday life, sensory information needs to be remembered both in the short- and long-term.

Hence, in the present study we followed-up the participants in our previous study and retested episodic memory for environmental sounds and odors. It should be noted that they were unaware that their memory would be tested a second time. The retention interval was approximately 1 year, an interval that would most likely lead to a substantial but not complete forgetting (e.g., Engen and Ross, 1973; Murphy et al., 1991), and for which it was reasonable to expect that most of the participants would be available for a retest. In line with our previous study, the familiarity of the odors and sounds was manipulated. In this way we could examine whether episodic recognition for auditory and olfactory information differed among early blind, late blind, and sighted participants and whether potential compensatory effects of blindness was influenced by stimulus familiarity. In addition, we assessed identification proficiency for the set of high familiar stimuli across the three study groups. Based on our previous study, we hypothesized that identification ability would be similar across the three groups. However, considering the limited number of episodic memory studies in the blind population, we had no hypotheses regarding potential differences in episodic long-term memory across the early blind, late blind, and sighted participants.

## Materials and Methods

### Participants

All participants from our previous study (Cornell Kärnekull et al., 2016) were asked to participate in a follow-up study with olfactory and auditory tasks. They were not informed that memory would be tested. From a total sample of 60 participants, 57 accepted to participate (i.e., 1 early blind and 2 late blind declined participation). Among these, 14 were early blind (age range: 29–67 years, mean age: 55.7 ± 11.3 years, 9 females), 13 late blind (age range: 45–74 years, mean age: 58.9 ± 10.7 years, 11 females), and 30 sighted individuals (age range: 25–75 years, mean age: 56.5 ± 12.4 years, 22 females). Each blind participant had an age- and sex-matched sighted participant. All participants reported that they had a normal sense of smell and hearing. Two participants were smokers.

The early blind participants were either congenitally blind or had become blind in early childhood (<2 years old), whereas the late blind participants had become blind in adulthood. Participant characteristics and self-reported causes and onset age of blindness are presented in **Table 1**.

**Table 1:** Blind participants’ group belonging and self-reported onset age of blindness, cause of blindness, and current visual acuity.

No.	Group	Self-reported onset age of blindness	Self-reported cause of blindness	Self-reported visual acuity
1	Early	congenital	Leber’s congenital amaurosis	totally blind
2	Early	1 year	Retinoblastom	totally blind
3	Early	2 years	Retinoblastom	totally blind
4	Early	congenital	Incontinentia pigmenti	totally blind
5	Early	2 wks	Retrolental fibroplasia	totally blind
6	Early	birth	Retrolental fibroplasia	totally blind
7	Early	3 months^∗^	Fetal infection (undiagnosed)	<0.05
8	Early	congenital	Heredo-retinopathia congenitalis	<0.05
9	Early	birth	Retrolental fibroplasia	<0.05
10	Early	congenital^∗^	Glaucoma	<0.05
11	Early	congenital^∗^	Axenfeld-Rieger syndrome	<0.05
12	Early	birth	Retrolental fibroplasia	<0.05
13	Early	congenital	Retinal degeneration	<0.05
14	Early	congenital	Leber’s congenital amaurosis	<0.05
15	Late	40 years	Retinitis pigmentosa	<0.05
16	Late	46 years	Retinitis pigmentosa	totally blind
17	Late	20 years	Glaucoma	totally blind
18	Late	62 years	Undetermined	<0.05
19	Late	57 years	Retinitis pigmentosa	<0.05
20	Late	58 years	Keratitis	totally blind
21	Late	38 years	Retinitis pigmentosa	totally blind
22	Late	51 years	Cataract, impaired cornea	<0.05
23	Late	29 years	Tumors pressing on the optic nerve	totally blind
24	Late	20 years	Stargardt’s disease	<0.05
25	Late	39 years	Tumors pressing on the optic nerve	<0.05
26	Late	50 years	Macular degeneration	<0.05
27	Late	28 years	Optic nerve inflammation	<0.05

*^∗^Participants reported they were born with visual acuity of less than 0.1 (legally blind).*

The time between initial testing (T1) and the follow-up (T2) varied between 399 and 644 days (*M* = 487, *SD* = 50, Median = 495). Importantly, the mean and median retention interval was similar for early blind (*M* = 509, *SD* = 57, Median = 525), late blind (*M* = 485, *SD* = 50, Median = 495), and sighted participants (*M* = 478, *SD* = 45, Median = 484). There was no statistically significant difference in the number of days retention interval between the groups (*F*_2,54_ = 1.90, *p* = 0.159).

The study was approved by the Regional Ethical Review Board in Stockholm (2015/369-31/4), and all participants provided written informed consent before the study. The participants were compensated for participating in the study (voucher à 400 SEK) and travel expenses for the blind participants were reimbursed.

### Materials

A total of 24 odors were used (**Table 2**), of which half had been presented at T1 and were now used as targets in the recognition test (6 high familiar and 6 low familiar). The other half of the odors comprised new distractors (6 high familiar and 6 low familiar). All odors were presented in felt-tip pens. Most of the high familiar odors pertained to the Sniffin’ Sticks identification tests (Hummel et al., 1997), whereas the selection of low familiar was based on previous studies (Cornell Kärnekull et al., 2015) and pilot studies. The low familiar odors were prepared in empty Sniffin’ Sticks and were hard to name (Cornell Kärnekull et al., 2016). The odors were rated for familiarity (see Procedure) on a 7-point scale (1 = *not familiar at all*, 7 = *very familiar*). A paired samples *t*-test confirmed that the high familiar odors (*M* = 5.1, *SD* = 0.9) were perceived as more familiar than the low familiar odors in the present study sample (*M* = 3.3, *SD* = 0.9), (*t*_56_ = 16.70, *p* < 0.001).

**Table 2:** Odor sets of high and low familiarity.

High familiar odors	Low familiar odors
Bitter almond^d^	1-Hexanol^b,e^
Clove^d^	2-Picoline^d^
Coffee^a,d^	Ethyl-diethyl malonate^b,e^
Garlic^a,e^	Heptanal^b,e^
Grass^a,e^	Leather^a,d^
Lilac^a,e^	Osmanthus^c,d^
Liquorice^a,e^	o-Toluidine^b,e^
Orange^a,e^	Starfleur^c,e^
Pineapple^a,d^	Styryl acetate^c,e^
Peach^a,e^	Violet leaf^c,d^
Smoked meat^a,d^	Walnut^d^
Thyme^d^	Ylang-ylang^d^

*^a^Selected from Sniffin’ Sticks Identification tests. ^b^Donated by the Department of Organic Chemistry at Stockholm University. ^c^International Flavors & Fragrances Inc. ^d^New distractor odors. ^e^Target odors.*

A total of 60 environmental sounds were used (**Table 3**), of which half were targets (15 high familiar and 15 low familiar) from the encoding session at T1 and half were new distractors (15 high familiar and 15 low familiar). The sounds were selected from a large sound database on CDs (BBC Sound Effects Library- Original Series, United Kingdom), from online collaborative sound databases (Freesound^1^ and SoundBible^2^), and from a study by Marcell, Borella, Greene, Kerr, and Rogers (Marcell et al., 2000). The duration of the sounds was set to 2 – 3 s. The selection of high and low familiar sounds was based on pilot studies in our lab. The sounds were rated for familiarity on a 7-point scale (1 = *not familiar at all*, 7 = *very familiar*). The participants rated the high familiar sounds as more familiar (*M* = 5.8, *SD* = 0.7) than the low familiar sounds (*M* = 3.7, *SD* = 0.8), (*t*_56_ = 25.02, *p* < 0.001).

**Table 3:** Sound sets of high and low familiarity.

High familiar sounds	Low familiar sound
Guitar^a^	Cat howling^a^
Pots clatter^a^	Shaking bottle of water^a^
Drink and swallow^a^	Pull out drawer^a^
Unlock and open a door^a^	Dishwasher^a^
Brushing teeth^a^	Coffee grinder^a^
Airplane^a^	Cat purr^a^
Blowing bubbles in water^a^	Welding^a^
Camera shot^a^	Push a chair^a^
Doorbell^a^	Cover a glass jar^a^
Dripping tap^a^	Phone^a^
Toilet flush^a^	Beveling on grinding wheel^a^
Goat^a^	Whip^a^
Squeaking bed^a^	Fart^a^
Whistle^a^	Sonar^a^
Air horn^a^	Stomach growl^a^
Seawash^b^	Wood fire^b^
Pulling a pint^b^	Fry egg^b^
Roulette wheel^b^	Cattle in hay^b^
Bread being sliced^b^	Gambling chip sorting machine^b^
Footsteps in snow^b^	Donkey walking past^b^
Horse trot^b^	Printing machinery^b^
Car started^b^	Pumping water by hand^b^
Inflating rubber dingy^b^	Ice skating spin^b^
Sail flapping^b^	Bicycle ride^b^
Car indicators^b^	Peeling an orange^b^
Eating a cracker^b^	Ice cube tray^b^
Lighter^b^	Stapler^b^
Paper rip^b^	Buttering a toast^b^
Cards shuffling^b^	Electric kettle^b^
Hair dryer^b^	Shaving cream^b^

*^a^New distractor sounds. ^b^Target sounds.*

A custom-build computer (OS: Microsoft Windows 7) that was connected to a soundcard (RMEHDSPe FX), D/A converter (RME ADI-8 QS), earphone amplifier (LP Phone-amp G109) and earphones (Beyerdynamic DT 990 Pro) was used for the presentation of sounds.

### Procedure

After being told about the general aim and procedure of the study, the participant provided written informed consent. The study comprised an olfactory session in a custom-made olfactory testing room with high-pressure ventilation and an auditory session in a custom-made and sound-isolated auditory testing room. The two test sessions were separated with a 20-min pause. Please note that the recognition test protocol in the present study was identical to Cornell Kärnekull et al. (2016), with the exception that it did not involve any learning phase of stimuli. The order of the olfactory and auditory sessions was identical to our previous study, with two exceptions due to practical circumstances (i.e., one of the lab rooms was occupied).

In short, the olfactory and auditory sessions consisted of a metacognitive judgment, an episodic recognition judgment, confidence, pleasantness, and familiarity ratings, and an identification task, respectively (in the stated order). Before the stimulus presentation, the participant made a metacognitive judgment (MJ) by estimating how many percent of the previously encoded stimulus material presented at the initial testing (T1) he/she thought would be recognized at the recognition test (cf. judgment of learning task in Cornell Kärnekull et al., 2016). Analyses of the metacognitive ability in predicting episodic odor and sound recognition performances are shown in Supplementary Figure S1 of the Supplementary material.

As mentioned above (Materials), the participant was presented with 24 odors and 60 environmental sounds at two separate sessions. After each presentation of an odor or sound, the participant made a yes/no judgment about whether or not the stimulus had been encoded at T1. Each recognition judgment was followed by a rating of confidence on a 5-point scale (1 = *not at all confident*, 3 = *moderately confident*, 5 = *very confident*) and ratings of perceived pleasantness (1 = *not pleasant at all*, 7 = *very pleasant*) and familiarity (1 = *not familiar at all*, 7 = *very familiar*) on 7-point scales. As a final task the participant was asked to identify the stimulus (e.g., coffee). The identification responses were dichotomously scored (1 = correct, 0 = incorrect). For odors, only the responses for the high familiar stimuli were scored. As noted in our previous study (Cornell Kärnekull et al., 2016), the reason for this was that the low familiar odors had been selected to be extremely hard to name, neither had a pre-defined corresponding name. Although all identification responses for the sounds were initially scored, for the sake of consistency only the high familiar sounds were analyzed in the present study. Hence, a total number of correct responses was calculated for high familiar odors (maximum = 12) and high familiar sounds (maximum = 30), respectively. However, please see Supplementary Table S1 in the Supplementary material for identification ability of low familiar odors and low familiar sounds.

For each sensory modality, a unique random presentation order was used for each pair of blind and matched sighted participant. All participants were blindfolded at testing. Please note that ratings of confidence, familiarity, and pleasantness across the three study groups are presented in the Supplementary material (Supplementary Figure S2).

### Data Analyses

Olfactory and auditory episodic recognition performance (*d′*) was analyzed for initial (T1) and follow-up (T2) testing in early blind, late blind, and sighted participants. *d′* served as an index of episodic recognition performance and is an unbiased measure of sensitivity (Macmillan and Creelman, 2005). In the signal detection theory model, *d′* is defined as the difference between *z*-transformed proportions of hits (H) and false alarms (FA); [*d*′ = *z* (H) – *z* (FA), where *z* is the inverse normal cumulative distribution function] (Macmillan and Creelman, 2005). Hit and false alarm rates of 1 and 0 were adjusted to 1 – 1/(2N) and 1/(2N), respectively, where N is the number of trials (Macmillan and Creelman, 2005). The data were collapsed across stimulus familiarity (low familiar, high familiar) since this factor did not interact with group (early blind, late blind, sighted) in explaining recognition performance at T2 (see Supplementary Table S2 and Supplementary Figure S3 in the Supplementary Material). We previously observed similar findings for memory after the short retention interval (Cornell Kärnekull et al., 2016). Hits and false alarms for odors and sounds at T2 are presented in the Supplementary material (Supplementary Figure S4). All analyses were conducted in SPSS and R (R Core Team, 2014).

## Results

**FIGURE 1:**
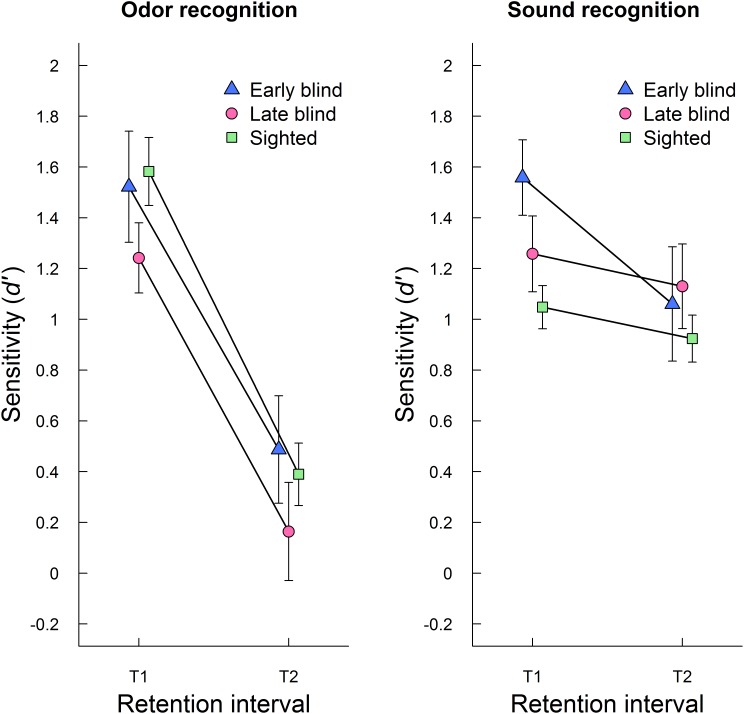
Aggregated episodic recognition performance (mean *d′* ± SE) for odors (left panel) and sounds (right panel) at initial testing (T1) and follow-up (T2) is plotted separately for early blind, late blind, and sighted participants.

In order to test the effect of blindness on auditory and olfactory episodic recognition as a function of retention interval, an ANOVA with group (early blind, late blind, sighted) as between-subjects factor and retention interval (T1, T2) and modality (odor, sound) as within-subjects factors was conducted. The results confirmed that the decrement in episodic recognition performance (*d′*) across retention intervals was significant (*F*_1,54_ = 73.32, *p* < 0.001, η^2^ = 0.26) and that memory was generally better for sounds than odors (*F*_1,54_ = 9.94, *p* = 0.003, η^2^= 0.04). There was no main effect of group (*F*_2,54_ = 0.99, *p* = 0.379, η^2^ = 0.04) or interaction between group and retention interval (*F*_2,54_ = 0.31, *p* = 0.733, η^2^ = 0.002). Retention interval and modality interacted, such that memory decrement was larger for odors than sounds (*F*_1,54_ = 35.18, *p* < 0.001, η^2^= 0.10). There was also an interaction between modality and group, (*F*_2,54_ = 3.49, *p* = 0.038, η^2^ = 0.03), indicating that early and late blind participants but not the sighted had better memory for sounds than odors. However, follow-up pairwise contrasts showed that the modality difference reached statistical significance only for late blind participants (*p* = 0.005). The three-way interaction between retention interval, group, and modality was not statistically significant (*F*_2,54_ = 1.34, *p* = 0.271, η^2^= 0.008). **Figure 1** shows mean recognition performance (*d′*) for each modality at T1 and T2, separately for early blind, late blind, and sighted participants. Although the three-way interaction was statistically non-significant, the figure illustrates that there is a smaller group difference in auditory memory at T2 than at T1. Further analysis of memory performance at T2 is presented below.

**FIGURE 2:**
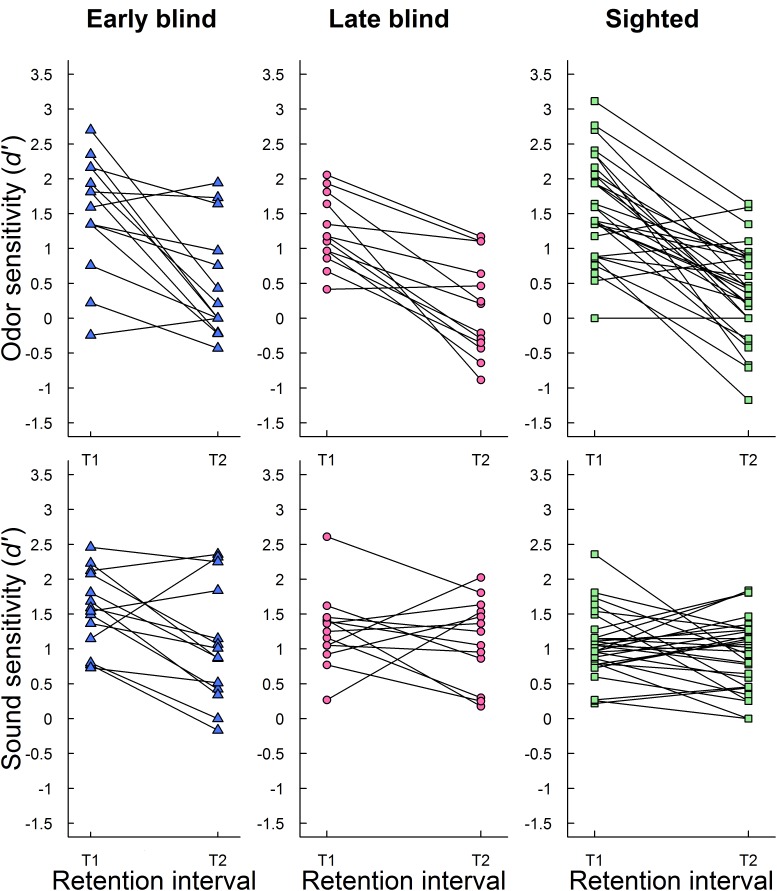
Individual episodic recognition performance (*d′*) for odors (upper panel) and sounds (lower panel) at initial testing (T1) and follow-up (T2) is plotted separately for early blind, late blind, and sighted participants.

To complement the picture of how recognition performance changed across time, individual recognition performances (*d′*) at T1 and T2 were inspected. **Figure 2** shows individual recognition performance for odors and sounds at T1 and T2 in early blind, late blind, and sighted participants. As expected, most participants had lower episodic recognition performance (*d′*) at follow-up (T2) than at initial testing (T1). However, there was some individual variation in performance across retention intervals. A few participants (*n* = 6; 2 early blind, 1 late blind, and 3 sighted) showed better odor memory at T2 than at T1, although the average increased number of correctly recognized odors was small (approximately 1.2 out of 24). A closer look at the underlying hits and false alarms showed that improvement was both related to more hits (*n* = 2) and fewer false alarms (*n* = 4). Similarly, a subsample of participants showed better memory for sounds at T2 than at T1 (*n* = 21; 6 early blind, 3 late blind, and 12 sighted). On average, they made 4.9 more correct recognition judgments out of 60 sounds. The improvements were related to both more hits (*n* = 7), fewer false alarms (*n* = 9), and a combination of the two (*n* = 5). The results are discussed below.

**FIGURE 3:**
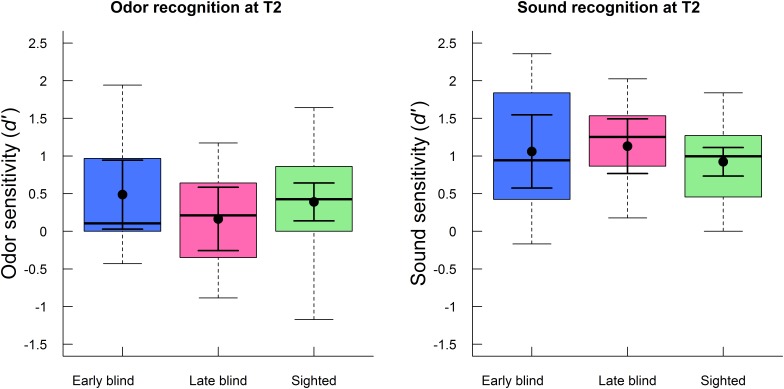
Boxplots of episodic recognition performance (*d′*) for odors (left panel) and sounds (right panel) at follow-up (T2) are displayed separately for early blind, late blind, and sighted participants. The boxes indicate the 25th, 50th (median), and 75th percentiles of the distribution (lower, middle, and upper horizontal lines of the box). The upper hinges indicate the maximum value of the variable located within a distance of 1.5 times the inter-quartile range above the 75th percentile. The lower hinges indicate the corresponding distance to the 25th percentile value. The means and 95% confidence intervals (dots and error bars in solid lines) are superimposed on the boxplots.

The effect of blindness on olfactory and auditory recognition performance (*d′*) at T2 specifically was analyzed with a mixed ANOVA, with group (early blind, late blind, sighted) as between-subjects factor and modality (odor, sound) as within-subjects factor. This analysis confirmed that there was no main effect of group (*F*_2,54_ = 0.29, *p* = 0.749, η^2^ = 0.01) or interaction between group and modality (*F*_2,54_ = 1.21, *p* = 0.307, η^2^ = 0.03) at the follow-up testing. In other words, olfactory performance was similar for early blind (*M* = 0.5, *SD* = 0.8), late blind (*M* = 0.2, *SD* = 0.7), and sighted (*M* = 0.4, *SD* = 0.7) participants. The group differences corresponded to approximately one more correctly recognized odor (out of 24) in early blind and sighted participants than in the late blind participants. As was the case for odor memory, recognition (*d′*) of environmental sounds was also similar for early blind (*M* = 1.1, *SD* = 0.8), late blind (*M* = 1.1, *SD* = 0.6), and sighted (*M* = 0.9, *SD* = 0.5) participants. The group differences corresponded to approximately one respective two more correctly recognized sounds (out of 60) in early blind and late blind participants than in sighted participants. There was a main effect of modality (*F*_1,54_ = 32.19, *p* < 0.001, η^2^ = 0.36), indicating that recognition performance at T2 was better for sounds (*M* = 1.0, *SD* = 0.6) than odors (*M* = 0.4, *SD* = 0.7). These *d′* values corresponded to approximately 40.5 correctly recognized sounds (*SD* = 6.0) out of 60 stimuli and 13.5 correctly recognized odors (*SD* = 2.9) out of 24. Both olfactory and auditory memory was better than what could be expected by chance (i.e., *d′* value of zero), as indicated by two one-sample *t*-tests (*t*_56_ = 3.87, *p* < 0.001 and *t*_56_ = 12.25, *p* < 0.001, respectively). Episodic recognition performance (*d′*) at T2 is presented as a function of group and modality in **Figure 3**.

**FIGURE 4:**
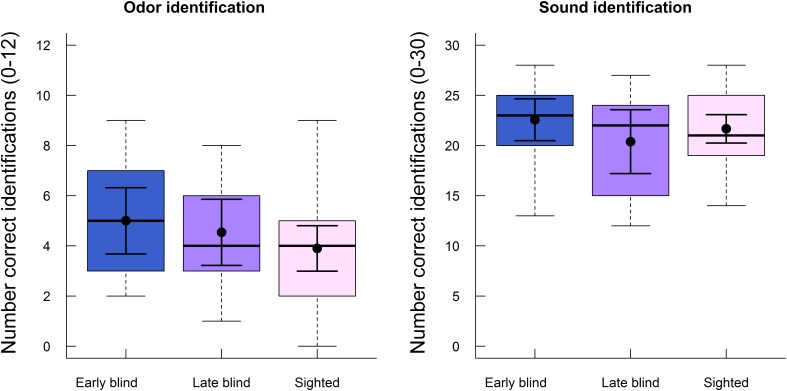
Boxplots of identification performance for high familiar odors (left panel) and high familiar sounds (right panel) at follow-up (T2) are displayed separately for early blind, late blind, and sighted participants. The boxes indicate the 25th, 50th (median), and 75th percentiles of the distribution (lower, middle, and upper horizontal lines of the box). The upper hinges indicate the maximum value of the variable located within a distance of 1.5 times the inter-quartile range above the 75th percentile. The lower hinges indicate the corresponding distance to the 25th percentile value. The means and 95% confidence intervals (dots and error bars in solid lines) are superimposed on the boxplots.

Lastly, we analyzed whether the ability to identify odors and sounds at T2 differed between the early blind, late blind, and sighted participants. As noted above, only high familiar stimuli were analyzed (see Methods section). As shown in **Figure 4**, identification of both odors and sounds was similar across the three groups. Performance was analyzed with a one-way ANOVA for each modality. The difference in the number of correctly identified stimuli (maximum score = 12) between early blind (*M* = 5.0, *SD* = 2.3), late blind (*M* = 4.5, *SD* = 2.2), and sighted (*M* = 3.9, *SD* = 2.4) participants was not statistically significant (*F*_2,54_ = 1.13, *p* = 0.331, η^2^ = 0.04). Similarly, the group differences observed for sound identification (maximum score = 30) between early blind (*M* = 22.6, *SD* = 3.6), late blind (*M* = 20.4, *SD* = 5.3) and sighted (*M* = 21.7, *SD* = 3.8) participants were relatively small and not statistically significant (*F*_2,54_ = 0.96, *p* = 0.391, η^2^ = 0.03). Please note that half of this material had also been presented at T1 and analyzed in our previous study (Cornell Kärnekull et al., 2016). Identification performance for the new stimuli only (i.e., distractors at T2) is presented in the Supplementary material (Supplementary Figure S5). An additional analysis on identification performance averaged across T1 and T2 suggested that there was no effect of blindness on identification of either odors or sounds (see Supplementary Table S3 in Supplementary Materials).

## Discussion

The present work investigated the effect of blindness on episodic memory for odors and sounds in the long-term. Memory performance was followed-up approximately 1 year after encoding and analyzed in relation to performance after a short retention interval (Cornell Kärnekull et al., 2016). For the short retention interval, we previously showed that the early blind participants had better episodic auditory memory than the sighted; a superiority that was absent at the follow-up assessment. As noted above (see section “Materials and Methods”), all analyses on episodic memory were performed on data collapsed across high and low familiar stimuli within respective modality. In general, recognition memory for sounds was better than for odors, and memory performance declined across the initial and follow-up assessments. However, the magnitude of decline was larger for olfactory than auditory information. Although this long retention interval is uncommon in olfactory research, memory performance above chance level after a similar time period has been shown previously in sighted individuals (Engen and Ross, 1973). To the best of our knowledge, episodic memory of environmental sounds has not been studied after this long retention interval before or been directly compared with odor memory in neither sighted nor blind individuals. However, in relation to other sensory stimuli (visual, tactile), memory of environmental sounds after relatively short retention intervals has been shown to be inferior (Lawrence et al., 1979; Cohen et al., 2009; Bigelow and Poremba, 2014).

As noted above, relatively few studies have investigated compensatory effects of blindness on episodic memory. Available evidence suggests that blind individuals have better memory for verbal and auditory information when memory is assessed shortly after stimulus presentation (Hötting and Röder, 2009, but see also Amedi et al., 2003 for more long-term assessment). In our previous study, we corroborated these observations as the early blind individuals showed better episodic memory for environmental sounds than the sighted after the short retention interval. However, in the present study where memory was assessed approximately 1 year after the first stimulus presentation, there were no longer any group differences in performance. This suggests that the auditory memory superiority among early blind may be prevalent after short retention intervals only. Our study is, to the best of our knowledge, the first report of a differential effect of blindness on long-term episodic memory across short and long retention intervals. As is discussed below, the lack of differences could not be explained by floor effects in performance at the follow-up assessment. Instead, this finding could potentially be related to the use of different memory strategies at the two test occasions. Although not explicitly tested in this study, we speculate that early blind participants might have attended to and encoded perceptual properties of the sounds better than the sighted, resulting in more accurate memory retrieval after the short retention interval. Research has shown that with time, memories may become more semantic and comprise of less contextual details (Harand et al., 2012). This could implicate that at the follow-up testing early blind participants had difficulties with remembering or making use of perceptual properties of the sounds for making correct recognition judgments. It is possible that after a longer time period, memory judgments were based on other factors, such as a more general feeling of familiarity of the stimuli presented. Rokem and Ahissar (2009) investigated the role of auditory perceptual acuity for verbal memory. Despite the obvious methodological differences between this study and ours, their findings should be considered. In their study, short-term memory recall (i.e., seconds) of pseudo words was initially better in congenitally blind than sighted participants, but when inter-subject variation in the participants’ thresholds was controlled for (i.e., word identification accuracy was kept equal) the group difference was eliminated. The authors suggested that blind individuals’ better memory capacity was related to better sensory encoding and consequently to better chunking of the words.

Most memory studies on compensatory effects of blindness have not been specifically designed to distinguish between underlying mechanisms of memory such as encoding, storage, and retrieval. However, Pasqualotto et al. (2013) used a false memory paradigm (i.e., Deese-Roediger-McDermott-paradigm) to study memory retrieval. In this study congenitally blind participants not only showed more accurate recall of encoded words but also less false memories than late blind and sighted. In a similar vein, Röder and Rösler (2003) showed that compared to sighted, congenitally blind participants showed both better recognition for environmental sounds (*d′*) and lower false memory rates for conceptually similar sounds to those presented during encoding (but only for one of the conditions). Although we never tested false memories in that sense, we have shown both in the present (see Supplementary material) and previous study (see Supplementary material of Cornell Kärnekull et al., 2016) that the early blind, late blind, and sighted participants had similar false alarms rates. Further research on episodic memory for perceptual information (e.g., sounds) is needed for explaining why episodic memory for environmental sounds may be better in blind than sighted individuals after a short but not long retention interval. One way to test the idea of better perceptual encoding in early blind individuals, as was mentioned above, would be to manipulate perceptual properties of environmental sounds (e.g., frequency, harmonics, and sound pressure level) so that targets and lures are conceptually identical but differ in perceptual features (see Röder and Rösler, 2003 for a similar approach). Recognition memory could be tested after different retention intervals with the hypothesis that early blind individuals would show better performance at shorter, but not longer, intervals. Having several test occasions would also enable to identify at what time intervals group differences are present or absent.

Memory performance of olfactory information showed another pattern of results. For this sensory modality, blindness was unrelated to memory performance both after the short and long retention intervals and consequently also to the rate of decline. This outcome suggests that episodic recognition for olfactory information is unrelated to blindness. Sorokowska and Karwowski (2017) recently showed similar findings for a large sample of blind and sighted participants who were tested shortly after encoding. It should be noted that although the effect of blindness on memory across time differed between the modalities, the interaction effect between retention interval, group, and modality was not statistically significant. Episodic odor recognition was relatively poor at the follow-up testing, although both olfactory and auditory memory performance was above chance level. Thus, the absence of group differences at the follow-up testing was not likely due to floor effects.

Moreover, in accordance with our previous study (Cornell Kärnekull et al., 2016), the early blind, late blind, and sighted were equally proficient at identifying odors and sounds. These findings support the notion that blindness has a minor influence on identification ability (e.g., Sorokowska, 2016). It should, however, be noted that previous research has yielded mixed evidence and that specific studies have reported enhanced odor identification ability in blind individuals (e.g., Cuevas et al., 2009).

Some methodological issues of the study need consideration. One limitation is the low statistical power following from limited sample sizes, which is common for studies involving blind participants. Furthermore, given the longitudinal study design, a different set of distractors was used at the follow-up assessment to minimize unwanted interference effects on memory from the first test occasion. However, it should be highlighted that the use of different distractors may potentially confound episodic memory performance as the distractors may vary in attributes that were not controlled for (e.g., variation in distinctiveness in relation to target stimuli). This could potentially explain why some participants had better memory at the follow-up than at initial testing. Nevertheless, we showed that the memory improvements were generally small and related to both more hits and less false alarms. The latter finding indicates that improvements cannot solely be due to more discriminable distractor items at follow-up.

## Conclusion

In conclusion the present work shows that there are no compensatory effects of blindness on episodic memory for environmental sounds and odors as assessed across long time frames. These observations contrast, in part, to the findings of our previous study on memory after a short retention interval in the same study sample, where early blind individuals had better memory for sounds than sighted. Regarding olfaction, our findings indicate that blindness may be unrelated to episodic odor memory after both short and long retention intervals. Taken together, these results demonstrate the need of using retention intervals with different lengths when studying the compensatory effects of blindness on memory performance.

## Data Accessibility

Data supporting this article is deposited at Open Science Framework: https://osf.io/pu4et/?view_only=f0a38f7d63ff4078827fa5749efb844f.

## Author Contributions

SCK participated in the conception and the design of the study, collected the data, carried out the statistical analyses, produced the figures, and drafted the manuscript. AA participated in the conception and in the design of the study, participated in the analysis of the data, and revised the manuscript. MN participated in the design of the study and in the analysis of the data, and revised the manuscript. ML participated in the design of the study and in the analysis of the data, and revised the manuscript. All authors gave final approval for publication.

## Conflict of Interest Statement

The authors declare that the research was conducted in the absence of any commercial or financial relationships that could be construed as a potential conflict of interest.

## Footnotes

1 www.freesound.org

2 www.soundbible.com

## References

[B1] Amedi A. Raz N. Pianka P. Malach R. Zohary E. (2003). Early “visual”cortex activation correlates with superior verbal memory performance in the blind. *Nat. Neurosci.* 6 758–766. 10.1038/nn1072 12808458

[B2] Araneda R. Renier L. A. Rombaux P. Cuevas I. De Volder A. G. (2016). Cortical plasticity and olfactory function in early blindness. *Front. Syst. Neurosci.* 10:75 10.3389/fnsys.2016.00075 PMC5003898 27625596

[B3] Bartlett J. C. (1977). Remembering environmental sounds: the role of verbalization at input. *Mem. Cogn.* 5 404–414. 10.3758/BF03197379 24203007

[B4] Bigelow J. Poremba A. (2014). Achilles’ Ear? inferior human short-term and recognition memory in the auditory modality. *PLoS One* 9:e89914. 10.1371/journal.pone.0089914 24587119 PMC3935966

[B5] Bower G. H. Holyoak K. (1973). Encoding and recognition memory for naturalistic sounds. *J. Exp. Psychol.* 101 360–366. 10.1037/h0035240 4753861

[B6] Cecchetti L. Kupers R. Ptito M. Pietrini P. Ricciardi E. (2016). Are supramodality and cross-modal plasticity the yin and yang of brain development? From blindness to rehabilitation. *Front. Syst. Neurosci.* 10:89. 10.3389/fnsys.2016.00089 27877116 PMC5099160

[B7] Cobb N. J. Lawrence D. M. Nelson N. D. (1979). Report on blind subject’s tactile and auditory recognition for environmental stimuli. *Percept. Mot. Skills* 48 363–366. 10.2466/pms.1979.48.2.363 461034

[B8] Cohen M. Horowitz T. Wolfe J. (2009). Auditory recognition memory is inferior to visual recognition memory. *Proc. Natl. Acad. Sci. U.S.A.* 106 6008–6010. 10.1073/pnas.0811884106 19307569 PMC2667065

[B9] R Core Team (2014). *R: A Language and Environment for Statistical Computing*. Vienna: R Foundation for Statistical Computing.

[B10] Cornell Kärnekull S. Arshamian A. Nilsson M. E. Larsson M. (2016). From perception to metacognition: auditory and olfactory functions in early blind, late blind, and sighted individuals. *Front. Psychol.* 7:1450. 10.3389/fpsyg.2016.01450 27729884 PMC5037222

[B11] Cornell Kärnekull S. Jönsson F. U. Willander J. Sikström S. Larsson M. (2015). Long-Term memory for odors: influences of familiarity and identification across 64 days. *Chem. Senses* 40 259–267. 10.1093/chemse/bjv003 25740304 PMC4398052

[B12] Cuevas I. Plaza P. Rombaux P. De Volder A. G. Renier L. (2009). Odour discrimination and identification are improved in early blindness. *Neuropsychologia* 47 3079–3083. 10.1016/j.neuropsychologia.2009.07.004 19616019

[B13] Engen T. Ross B. M. (1973). Long-term memory of odors with and without verbal descriptions. *J. Exp. Psychol.* 100 221–227. 10.1037/h0035492 4745452

[B14] Frankland P. W. Bontempi B. (2005). The organization of recent and remote memories. *Nat. Rev. Neurosci.* 6 119–130. 10.1038/nrn1607 15685217

[B15] Gougoux F. Lepore F. Lassonde M. Voss P. Zatorre R. J. Belin P. (2004). Neuropsychology: pitch discrimination in the early blind. *Nature* 430 309–309. 10.1038/430309a 15254527

[B16] Harand C. Bertran F. La Joie R. Landeau B. Mézenge F. Desgranges B. (2012). The hippocampus remains activated over the long term for the retrieval of truly episodic memories. *PLoS One* 7:e43495. 10.1371/journal.pone.0043495 22937055 PMC3427359

[B17] Hötting K. Röder B. (2009). Auditory and auditory-tactile processing in congenitally blind humans. *Hear. Res.* 258 165–174. 10.1016/j.heares.2009.07.012 19651199

[B18] Hummel T. Sekinger B. Wolf S. R. Pauli E. Kobal G. (1997). “Sniffin” sticks’: olfactory performance assessed by the combined testing of odor identification, odor discrimination and olfactory threshold. *Chem. Senses* 22 39–52. 10.1093/chemse/22.1.39 9056084

[B19] Kandel E. R. Dudai Y. Mayford M. R. (2014). The molecular and systems biology of memory. *Cell* 157 163–186. 10.1016/j.cell.2014.03.001 24679534

[B20] Kupers R. Ptito M. (2014). Compensatory plasticity and cross-modal reorganization following early visual deprivation. *Neurosci. Biobehav. Rev.* 41 36–52. 10.1016/j.neubiorev.2013.08.001 23954750

[B21] Lawless H. T. (1978). Recognition of common odors, pictures, and simple shapes. *Percept. Psychophys.* 24 493–495. 10.3758/BF03198772 750989

[B22] Lawless H. T. Cain W. S. (1975). Recognition memory for odors. *Chem. Senses* 1 331–337. 10.1093/chemse/1.3.331

[B23] Lawrence D. M. Banks W. P. (1973). Accuracy of recognition memory for common sounds. *Bull. Psychon. Soc.* 1 298–300. 10.3758/BF03334350

[B24] Lawrence D. M. Cobb N. J. Beard J. I. (1979). Comparison of accuracy in auditory and tactile recognition memory for environmental stimuli. *Percept. Mot. Skills* 48 63–66. 10.2466/pms.1979.48.1.63 450641

[B25] Macmillan N. A. Creelman C. D. (2005). *Detection Theory: A user’s Guide*, 2nd Edn. Mahwah, NJ: Erlbaum.

[B26] Marcell M. M. Borella D. Greene M. Kerr E. Rogers S. (2000). Confrontation naming of environmental sounds. *J. Clin. Exp. Neuropsychol.* 22 830–864. 10.1076/jcen.22.6.830.949 11320440

[B27] Merabet L. B. Pascual-Leone A. (2010). Neural reorganization following sensory loss: the opportunity of change. *Nat. Rev. Neurosci.* 11 44–52. 10.1038/nrn2758 19935836 PMC3898172

[B28] Murphy C. Cain W. S. Gilmore M. M. Skinner R. B. (1991). Sensory and semantic factors in recognition memory for odors and graphic stimuli: elderly versus young persons. *Am. J. Psychol.* 104 161–192. 10.2307/1423153 1862819

[B29] Occelli V. Lacey S. Stephens C. Sathian K. (2016). Superior verbal abilities in congenital blindness. *Electron. Imaging* 2016 1–4. 10.2352/ISSN.2470-1173.2016.16.HVEI-094

[B30] Occelli V. Spence C. Zampini M. (2013). Auditory, tactile, and audiotactile information processing following visual deprivation. *Psychol. Bull.* 139 189–212. 10.1037/a0028416 22612281

[B31] Pasqualotto A. Lam J. S. Proulx M. J. (2013). Congenital blindness improves semantic and episodic memory. *Behav. Brain Res.* 244 162–165. 10.1016/j.bbr.2013.02.005 23416237

[B32] Pasqualotto A. Proulx M. J. (2012). The role of visual experience for the neural basis of spatial cognition. *Neurosci. Biobehav. Rev.* 36 1179–1187. 10.1016/j.neubiorev.2012.01.008 22330729

[B33] Raz N. Striem E. Pundak G. Orlov T. Zohary E. (2007). Superior serial memory in the blind: a case of cognitive compensatory adjustment. *Curr. Biol.* 17 1129–1133. 10.1016/j.cub.2007.05.060 17583507

[B34] Röder B. Rösler F. (2003). Memory for environmental sounds in sighted, congenitally blind and late blind adults: evidence for cross-modal compensation. *Int. J. Psychophysiol.* 50 27–39. 10.1016/S0167-8760(03)00122-3 14511834

[B35] Röder B. Rösler F. Neville H. J. (2001). Auditory memory in congenitally blind adults: a behavioral-electrophysiological investigation. *Cogn. Brain Res.* 11 289–303. 10.1016/S0926-6410(01)00002-7 11275490

[B36] Rokem A. Ahissar M. (2009). Interactions of cognitive and auditory abilities in congenitally blind individuals. *Neuropsychologia* 47 843–848. 10.1016/j.neuropsychologia.2008.12.017 19138693

[B37] Sorokowska A. (2016). Olfactory performance in a large sample of early-blind and late-blind individuals. *Chem. Senses* 41 703–709. 10.1093/chemse/bjw081 27439429

[B38] Sorokowska A. Karwowski M. (2017). No sensory compensation for olfactory memory: differences between blind and sighted people. *Front. Psychol.* 8:2127. 10.3389/fpsyg.2017.02127 29276494 PMC5727095

[B39] Wan C. Y. Wood A. G. Reutens D. C. Wilson S. J. (2010). Early but not late-blindness leads to enhanced auditory perception. *Neuropsychologia* 48 344–348. 10.1016/j.neuropsychologia.2009.08.016 19703481

