# Supplementary material for: The Effect of Blindness on Long-Term Episodic Memory for Odors and Sounds
Source: Front Psychol. 2018 Jun 20;9:1003. doi: 10.3389/fpsyg.2018.01003 (PMC6020764; doi:10.3389/fpsyg.2018.01003)
Supplement: Supplementary file 1 [file Table_1.PDF]

Supplementary Material

The effect of blindness on long-term episodic memory of odors and sounds

Stina Cornell Kärnekull[1*], Artin Arshamian[1,2,3], Mats E Nilsson[1], Maria Larsson[1]
[*] Correspondence: Stina Cornell Kärnekull: stina.cornell.karnekull@psychology.su.se

Table S1. Identification performance (mean number of correct responses) is presented for low familiar sounds (maximum score = 30) and low familiar odors (maximum score = 12) at T2 as a function of group (early blind, late blind, sighted). The standard deviations are presented within the parentheses.

	Identification	
	Low familiar sounds	Low familiar odors
Early blind	6.6 (2.7)	0.07 (0.3)
Late blind	5.7 (3.0)	0.08 (0.3)
Sighted	5.3 (3.0)	0.07 (0.3)
