# Supplementary material for: The Effect of Blindness on Long-Term Episodic Memory for Odors and Sounds
Source: Front Psychol. 2018 Jun 20;9:1003. doi: 10.3389/fpsyg.2018.01003 (PMC6020764; doi:10.3389/fpsyg.2018.01003)
Supplement: Supplementary file 2 [file Table_2.PDF]

Supplementary Material

The effect of blindness on long-term episodic memory of odors and sounds

Stina Cornell Kärnekull[1*], Artin Arshamian[1,2,3], Mats E Nilsson[1], Maria Larsson[1]
[*] Correspondence: Stina Cornell Kärnekull: stina.cornell.karnekull@psychology.su.se

Table S2. Results from separate univariate analyses of variance (ANOVAs) on sensitivity (*d'*) at follow-up with familiarity (high, low) and group (early blind, late blind, sighted) as independent variables are presented for odors and sounds, respectively.

Modality	Dependent variable	Independent variable	*F*	*df*	*p*	η^2
Odor	Sensitivity (*d'*)	Group	0.76	2,54	.472	.027
		Familiarity	0.30	1,54	.588	.005
		Group * Familiarity	1.02	2,54	.367	.036
Sound	Sensitivity (*d'*)	Group	0.57	2,54	.568	.021
		Familiarity	6.44*	1,54	.014	.106
		Group * Familiarity	0.22	2,54	.805	.007

Note. $^{*}p < 0.05$
