# Supplementary material for: The Effect of Blindness on Long-Term Episodic Memory for Odors and Sounds
Source: Front Psychol. 2018 Jun 20;9:1003. doi: 10.3389/fpsyg.2018.01003 (PMC6020764; doi:10.3389/fpsyg.2018.01003)
Supplement: Supplementary file 3 [file Table_3.PDF]

Supplementary Material

The effect of blindness on long-term episodic memory of odors and sounds

Stina Cornell Kärnekull[1*], Artin Arshamian[1,2,3], Mats E Nilsson[1], Maria Larsson[1]
[*] Correspondence: Stina Cornell Kärnekull: stina.cornell.karnekull@psychology.su.se

Table S3. Results from separate univariate one-way analyses of variance (ANOVAs) on averaged identification performance at initial (T1) and follow-up (T2) testing are presented for odors and sounds, respectively. Group (early blind, late blind, sighted) is the independent variable.

Modality	Dependent variable	Independent variable	*F*	*df*	*p*	η^2
Odor	Identification	Group	2.25	2,54	.115	.077
Sound	Identification	Group	0.79	2,54	.457	.029
