# Supplementary material for: The Effect of Blindness on Long-Term Episodic Memory for Odors and Sounds
Source: Front Psychol. 2018 Jun 20;9:1003. doi: 10.3389/fpsyg.2018.01003 (PMC6020764; doi:10.3389/fpsyg.2018.01003)
Supplement: Supplementary file 4 [file Image_1.PDF]

# *Supplementary Material*

## The effect of blindness on long-term episodic memory of odors and sounds

**Stina Cornell Kärnekull[1*], Artin Arshamian[1,2,3], Mats E Nilsson[1], Maria Larsson[1]**
[*] Correspondence: Stina Cornell Kärnekull: stina.cornell.karnekull@psychology.su.se

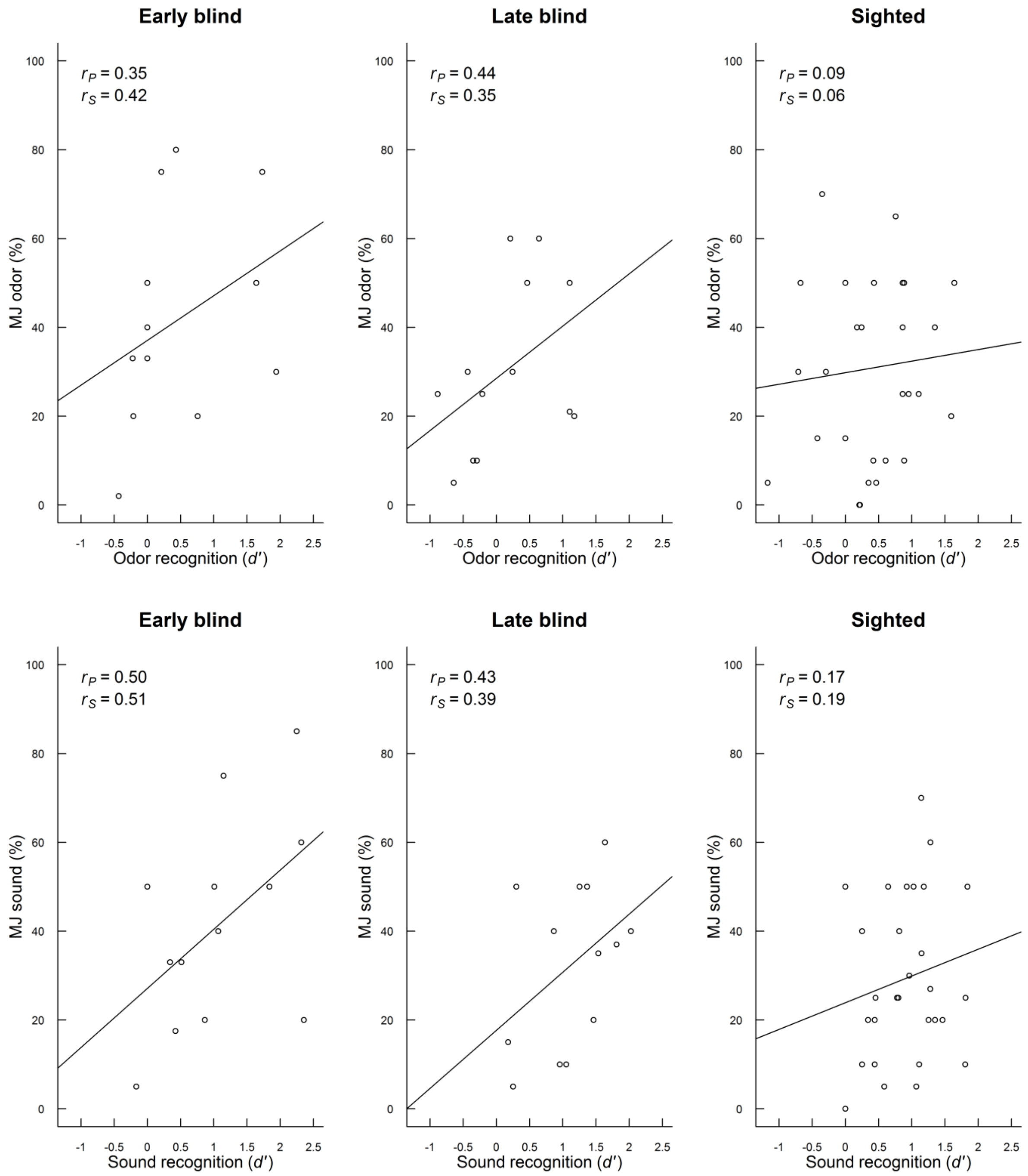


**Figure S1.** Correlations between metacognitive judgment (MJ) and episodic recognition ($d'$) at follow-up are plotted for odors (upper panel) and sounds (lower panel), separately for early blind, late blind, and sighted participants. The Pearson product-moment correlation coefficient

($r_P$), with fitted regression line (ordinary least squares), and the Spearman's rank correlation coefficient ($r_S$) are depicted. None of the correlation coefficients reached statistical significance.
