# Supplementary material for: The Effect of Blindness on Long-Term Episodic Memory for Odors and Sounds
Source: Front Psychol. 2018 Jun 20;9:1003. doi: 10.3389/fpsyg.2018.01003 (PMC6020764; doi:10.3389/fpsyg.2018.01003)
Supplement: Supplementary file 6 [file Image_3.PDF]

Supplementary Material

The effect of blindness on long-term episodic memory of odors and sounds

Stina Cornell Kärnekull[1*], Artin Arshamian[1,2,3], Mats E Nilsson[1], Maria Larsson[1]
[*] Correspondence: Stina Cornell Kärnekull: stina.cornell.karnekull@psychology.su.se

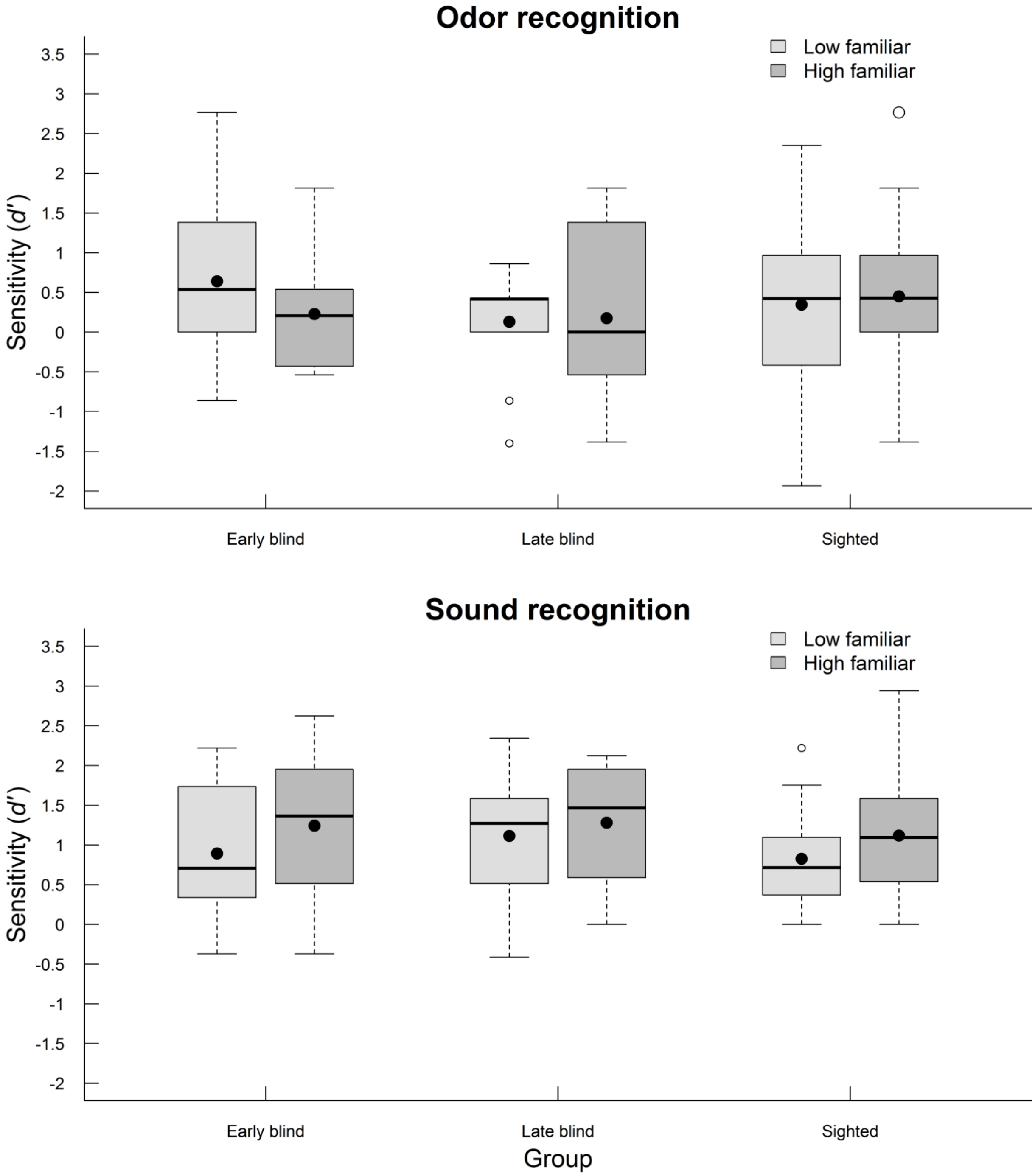

Figure S3. Boxplots of episodic recognition (*d'*) of odors (upper panel) and sounds (lower panel) at follow-up are displayed separately for low familiar (light grey boxes) and high familiar (dark grey boxes) stimuli in early blind, late blind, and sighted participants. The

boxes indicate the 25th, 50th (median), and 75th percentiles of the distribution (lower, middle, and upper horizontal lines of the box). The upper hinges indicate the maximum value of the variable located within a distance of 1.5 times the inter-quartile range above the 75th percentile. The lower hinges indicate the corresponding distance to the 25th percentile value. Circles indicate values outside these hinges (outliers). The means (dots) are superimposed on the boxplots.
